# CSF-1R inhibitor PLX3397 attenuates peripheral and brain chronic GVHD and improves functional outcomes in mice

**DOI:** 10.1186/s12974-023-02984-7

**Published:** 2023-12-15

**Authors:** Samreen N. Shaikh, Emily F. Willis, Max Dierich, Yi Xu, Samuel J. S. Stuart, Glenda C. Gobe, Abate A. Bashaw, Oliver Rawashdeh, Seung Jae Kim, Jana Vukovic

**Affiliations:** 1 School of Biomedical Sciences, Faculty of Medicine, The University of Queensland, St Lucia, Brisbane, QLD 4072 Australia; 2 Queensland Brain Institute, The University of Queensland, Brisbane, QLD Australia

**Keywords:** Bone-marrow transplant, Chronic GVHD, Macrophage depletion, Colony stimulating factor-1 receptor, Cognitive dysfunction

## Abstract

Graft-versus-host disease (GVHD) is a serious complication of otherwise curative allogeneic haematopoietic stem cell transplants. Chronic GVHD induces pathological changes in peripheral organs as well as the brain and is a frequent cause of late morbidity and death after bone-marrow transplantation. In the periphery, bone-marrow-derived macrophages are key drivers of pathology, but recent evidence suggests that these cells also infiltrate into cGVHD-affected brains. Microglia are also persistently activated in the cGVHD-affected brain. To understand the involvement of these myeloid cell populations in the development and/or progression of cGVHD pathology, we here utilized the blood–brain-barrier permeable colony stimulating factor-1 receptor (CSF-1R) inhibitor PLX3397 (pexidartinib) at varying doses to pharmacologically deplete both cell types. We demonstrate that PLX3397 treatment during the development of cGVHD (i.e., 30 days post-transplant) improves disease symptoms, reducing both the clinical scores and histopathology of multiple cGVHD target organs, including the sequestration of T cells in cGVHD-affected skin tissue. Cognitive impairments associated with cGVHD and neuroinflammation were also attenuated by PLX3397 treatment. PLX3397 treatment prior to the onset of cGVHD (i.e., immediately post-transplant) did not change in clinical scores or histopathology. Overall, our data demonstrate significant benefits of using PLX3397 for the treatment of cGVHD and associated organ pathologies in both the periphery and brain, highlighting the therapeutic potential of pexidartinib for this condition.

**Supplementary Information:**

The online version contains supplementary material available at 10.1186/s12974-023-02984-7.

## Key points

- PLX3397 treatment during the progression phase of chronic graft-versus-host disease (cGVHD) attenuates pathology in multiple organ systems and reduces T-cell infiltration into the skin.
- PLX3397 treatment during cGVHD progression attenuates microglia/macrophage reactivity and major histocompatibility complex class II (MHCII) expression in the brain.
- PLX3397 treatment during cGVHD progression alleviates cognitive impairment.

**Supplementary Information:**

The online version contains supplementary material available at 10.1186/s12974-023-02984-7.

## Introduction

Allogeneic stem cell transplantation has therapeutic benefits for patients with haematological malignancies but may give rise to graft-versus-host disease (GVHD). GVHD develops when allogeneic T cells in the donor graft, intended to kill residual cancer cells, instead initiate an immune attack on the recipient’s host cells [1]. GVHD is divided into acute and chronic variants, with tissue injury and subsequent inflammation triggered by the conditioning regime being the initiating factors for both forms of GVHD [2]. Although the organs affected by acute GVHD (aGVHD) are generally limited [3], chronic GVHD (cGVHD) has a widespread effect in different organs and is, therefore, seriously debilitating in nature [4, 5]. Prominent features of cGVHD pathophysiology are inflammation and fibrosis of peripheral organs [6]. Skin is primarily affected by cGVHD, with clinical manifestations including lichen planus and sclerotic effects leading to significant functional disability [2]. Other organs also undergo pathological change in cGVHD, such as bronchiolitis obliterans in the lungs, fibrosis of the liver, and ulcerations in the gastrointestinal tract [2, 7], all of which are associated with significant morbidity and mortality. In the central nervous system (CNS), GVHD manifests as neurocognitive dysfunction in up to 60% of patients [8]. Statistically, up to 70% of transplant survivors who develop cGVHD experience severe detriments to their post-transplant quality of life [9, 10]. Unfortunately, therapeutic options to treat cGVHD are limited, and there is thus a pressing need for effective therapies in cGVHD treatment. Ideally, such therapies would treat both the peripheral as well neurocognitive deficits induced by cGVHD to improve patient’s quality of life.

The mechanisms of cGVHD pathogenesis are not well-understood. Adaptive and innate immune cells, including B cells, T_reg_ cells, natural killer T cells, dendritic cells, and innate lymphoid cells, are all involved in cGVHD [11, 12]. Macrophage infiltration in particular appears a common feature of cGVHD pathologies. Recently, we reported that macrophage presence not only associates with the peripheral pathologies but also CNS-associated pathologies of cGVHD [13]. We showed that there is infiltration of donor macrophages (expressing major histocompatibility complex class II (MHCII)) into the brain during cGVHD, which correlated with cognitive dysfunction and neuroinflammation [13]. These donor macrophages maintained a distinct transcriptional identity compared to host microglia in cGVHD-affected brains, but the latter population nonetheless appeared activated and there were concurrent observations of disrupted synaptic connectivity. The functional significance of this microglial activation, and also the presence of peripheral macrophages, in the manifestation and/or progression of cGVHD-associated brain pathology is unclear. Addressing this issue may aid the development of new therapies that can simultaneously target both peripheral macrophages and CNS-resident microglia to improve patient outcomes.

One approach for the pharmacologic targeting of microglia and macrophages is offered by their critical dependence on the type III tyrosine kinase receptor, CSF-1R, for survival and turnover [14–16]. A recently developed CSF-1R inhibitor, PLX3397 (or Pexidartinib), which is orally potent and can cross the blood–brain barrier, is currently designated as a ‘Breakthrough Therapy’ by the FDA for the treatment of tenosynovial giant cell tumour (TGCT) and other solid tumours [17]. Due to its ability to cross the BBB and target microglia, we here investigated whether PLX3397 could be repurposed as a macrophage/microglia depletion therapy to improve both the peripheral and CNS outcomes of cGVHD in mice.

## Methods

### Mice

Eight-to-thirteen-week-old female C57BL/6 and B6D2F1 mice were used for this study. All mice were housed (2–5 mice per cage) in individually ventilated cages on a 12 h light/dark cycle with ad libitum access to food and water*.* Mice were sourced from Animal Resource Centre (Canning Vale, Western Australia). All experimental procedures were conducted in accordance with the Australian Code for the Care and Use of Animals for Scientific Purposes with approval from The University of Queensland Animal Ethics Committee.

### Irradiation and graft preparation

Irradiation and cell transplants were conducted as previously described [18–20]. This conditioning and cell transplantation model results in successful immune cell chimerism [19]. Briefly, 1 day prior to transplantation, recipient B6D2F1 mice received 1100 centigray (cGy) of total body irradiation (Gamma cell irradiator), split into two doses that were administered 3 h apart to minimise gastrointestinal toxicity. The following day, each recipient B6D2F1 mouse received a tail vein injection containing 5 × 10^6^ bone-marrow cells. Following transplantation, all mice were housed in sterilised cages and received autoclaved food and water.

The ‘syngeneic’ or the non-cGVHD control group received a cell transplant from donor B6D2F1 mice. For the allogeneic transplant (the ‘cGVHD’ group), B6D2F1 recipients received cells from donor C57BL/6 mice. In allotransplant recipients, the bone-marrow mixture was also supplemented with 0.5 × 10^6^ mature T cells, isolated from whole spleen and using magnetic bead-mediated depletion to remove non-T cells. Briefly, whole spleen was mashed, followed by red cell lysis. Next, the single-cell splenocyte suspension was incubated with a cocktail of mAb CD19 [HB305], CD11b [TIB128], anti-B220 [RQ36B2], anti-Gr1 [RB6-8C5], and anti-Ly76 [TER119]) for 20 min on ice. Cells were subsequently resuspended in goat anti-rat IgG BioMag beads (QIAGEN) and incubated again for 20 min on ice, followed by depletion of antibody-bound cells using magnetic separator. Non-bound T cells were stained with anti-CD3-BV421 (Biolegend) and confirmed to be 80–90% pure.

### PLX3397 treatment

PLX3397 was provided and used under the permission of a material transfer agreement from Plexxikon (USA), and incorporated into AIN-76A mouse chow at either 150 parts per million (ppm) or 300 ppm, provided via Research Diets (USA, NJ). Control mice received standard chow (AIN-76A, Research Diets). The timings of each chow’s administration were relative to the bone-marrow transplantation, and indicated on the experimental timelines within the figures.

### Clinical scoring to assess GVHD development

Transplanted animals were monitored daily and assessed weekly for the degree of systemic GVHD, using a scoring system based on five clinical parameters: weight loss, activity, posture (hunching), fur texture and skin integrity [21] (Table 1). Each animal was graded from 0 to 2 for each criterion, with a final score assigned by adding the grades of all five parameters; assessors were blinded to the experimental group and/or treatment condition. Animals with a cumulative GVHD score of six or above were euthanized, and the next day recorded as the day of death, in accordance with the guidelines of the institutional animal ethics committee.

**Table 1:** Animal monitoring sheet for GVHD

Criteria	0	0.5	1.0	1.5	2.0
Skin integrity	Normal	Minor scaling of paws OR tail OR ears only	Scaling of paws and tail	Areas of hair loss with skin thickening	Obvious areas of denuded skin
Posture (hunching)	Normal	Very minor hunching, apparent at rest only	Clear hunching apparent at rest only	Clear hunching, apparent with movement	Severe hunching, impairs movement
Fur texture	Normal	Minor/partial ruffling over ventral surface only	Mild to moderate ruffling over ventral surface	Moderate/complete ruffling over ventral surface and partial ruffling over dorsal surface	Severe/complete ruffling over ventral and dorsal surface, poor grooming
Activity	Normal	Minor reduction only	Mild to moderate reduction	Moderate to severe reduction	Stationary unless stimulated
Weight loss	< 10%		10–25%		25%

### Active place avoidance cognitive test

To assess spatial learning and memory, all animals underwent testing in active place avoidance (APA) test, starting on day 70 post-transplant, as described previously [22]. Briefly, the APA apparatus consisted of a rotating circular arena with a 60° shock zone, the location of which remained fixed relative to the room coordinates. Distinct black-and-white A3 size visual cues were located on the walls of the room. During testing, entry into the shock zone led to the delivery of a brief foot shock. Mice were tasked with actively avoiding the shock zone using the visual cues located around the room. On day 0 of testing, mice were habituated by being placed in the rotating arena and allowed to explore freely with the shock zone turned off. Next, mice were tested for 20 min each day for five consecutive days with shock turned on. Throughout the testing period, the position of the animal in the arena was tracked using an overhead camera linked to Tracker software (Bio-Signal Group, version 2.36). Once the trials were completed, the recorded tracks for each mouse were analysed using Track Analysis (Bio-Signal Group, version 2.2). We found that naive mice on either control chow or 300 ppm PLX3397 chow were comparable to each other in terms of their percent improvement in the APA task (i.e., shock zone entries on day 5 compared to day 1 of testing) (Fig. 3D), and these groups were, therefore, later combined to represent a single naive group.

### TSE PhenoMaster assessment and voluntary wheel running activity

Prior to the beginning of PhenoMaster testing, all animals were first individually housed for a week in environmentally controlled acclimatisation chambers. The acclimatisation chambers were maintained at a 12 h light:dark cycle, a temperature of 25 °C and humidity level of 50%. After 1 week of acclimatisation, mice were weighed and moved into PhenoMaster cabinets for 1 week of testing, during which mice have ad libitum access to food and water that is in turn measured with sensors, and the overall consumption is calculated at the end of 1 week. Locomotor activity of mice was measured by two infrared sensor frames located on top of one another, such that the bottom one records horizontal movement in *x*–*y* plane, such as walking or running, whereas the upper one measures vertical movement in *z* plane-like rearing. Mouse movement was recorded based on the light beam breaks in a particular time duration required, and physical activity was calculated as the total number of rearing events (*z* axis) plus fine and ambulatory movements (*x*–*y* plane). Cabinets were equipped with O_2_/CO_2_ sensors for measuring respiratory exchange ratios and energy consumption.

PhenoMaster cages were also equipped with running wheels provided ad libitum to mice for the duration of the testing period. Running wheels were 11.5 cm and 4 cm in diameter and width, respectively, and were made of stainless steel. The PhenoMaster software continuously records running wheel parameters such as maximum run distance covered, maximum run speed, average run speed, run time, run attempts, and running wheel turns for the entire duration of testing. After completion of the testing period, running wheel activity for each mouse was calculated as the sum of left and right running wheel turns on days 5, 6 and 7 of testing.

### Tissue harvesting and processing

Mice were euthanised using intraperitoneal (i.*p*.) injection of sodium pentobarbital (1.6 mg/g body weight) prior to transcardial perfusion with 1 × phosphate buffered saline (PBS) and 10% formalin. GVHD target tissues (skin, lung, liver and gut) were dissected out, fixed in formalin for 24 h, dehydrated in increasing concentrations of ethanol, followed by clearing in xylene and embedding in paraffin. Tissues were sectioned into 5 μm-thick sections using a rotary microtome (Thermofisher) and mounted on glass slides for staining. For fixed brain specimens, free-floating sections (40 µm) were obtained on a sliding microtome (Leica) and collected in 1 × PBS with 0.05% sodium azide until further processing.

### Histopathology scoring

For pathology scoring of GVHD target organs, all tissues were deparaffinized in xylene and then rehydrated through immersion in aqueous solutions with a decreasing ethanol concentration. This was then followed by haematoxylin and eosin (H&E) staining, dehydration in ethanol mounting and coverslipping. Imaging was conducted using the Metafer Vslide Scanner (MetaSystems) with a Zeiss Axio Imager Z2 at a magnification of 20X. Sections were examined with the help of a qualified pathologist (G.C.G) in a blinded fashion using an established semiquantitative scoring system for GVHD (Table 2) [23]. In brief, for skin, a grade of one was assigned if lymphocyte infiltration associated with small apoptotic regions was observed. Higher scores were primarily determined by the level of separation of the dermis and epidermis. The presence of small, sporadic and localised regions of separation was classed as grade two, while larger widespread regions were classed as grade three, and a near-complete separation of the dermis and epidermis as grade four. For both the lungs and liver, the primary criterion for scoring was the degree to which mononuclear cells infiltrated the parenchyma of the organs, near the bronchi (lungs) or blood vessels (liver). For a grade of one, a small number of mononuclear cells were observed to be infiltrating near the bronchi or vessels; for a grade of two, mononuclear infiltration was widespread; for a grade of three, both widespread peri-bronchial/vascular infiltration and localised infiltration into the parenchyma of the organs was present. A grade of four was assigned when a large degree of infiltration into the parenchyma was observed. For the gut, a grade of one was assigned if necrosis of epithelial cells was observed. If necrosis of glands was also observed, a grade of two was assigned. Higher grades were scored in the presence of localised (grade three) or widespread (grade four) mucosal denudation.

**Table 2:** Grading criteria for pathology in peripheral tissues

Grade	Skin	Liver	Lung	Gut
0	No visible pathology	No visible pathology	No visible pathology	No visible pathology
I	Low degree of apoptosis, lymphocyte infiltration	Punctate infiltration of mononuclear cells	Punctate infiltration of mononuclear cells	Single cell necrosis of epithelial cells
II	Focal separation of the dermis and epidermis	Sporadic perivascular infiltration of mononuclear cells	Sporadic clustering of mononuclear cells, particularly clustering around bronchi	Necrosis and loss of glands
III	Widespread separation of the dermis and epidermis	Sporadic perivascular infiltration of mononuclear cells with infiltration into the parenchyma	Clustering of mononuclear cells, some loss of eosin staining on bronchi	Focal microscopic mucosal denudation
IV	Complete or near complete separation of the dermis and epidermis	Widespread perivascular infiltration of mononuclear cells with spread into the parenchyma	Clustering of mononuclear cells with infiltration into the parenchyma, loss of structural integrity in bronchi	Diffuse mucosal denudation

### Masson’s trichrome staining and fibrosis scoring

To assess skin fibrosis, paraffin embedded skin sections were collected and deparaffinized, as described above and Masson’s trichrome stained. Slides were then fixed in Bouin’s solution for 1 h at 56 °C and then rinsed in water for 10 min. Slides were stained in Weigert’s iron hematoxylin working solution for 10 min (1 g hematoxylin in 100 mL 95% ethanol added to 4 mL 29% ferric chloride with 1 mL hydrochloric acid in 95 mL distilled water). Slides were washed and stained in Bierich scarlet-acid fuchsin solution (90 mL 1% aqueous Biebrich scarlet, 10 mL 1% aqueous acid fuchsin and 1 mL glacial acetic acid) for 10 min then washed in distilled water and differentiated in phosphomolybdic–phosphotungstic acid solution for 7 min (25 mL 5% phosphomolybdic acid, 25 mL 5% phosphotungstic acid). Slides were stained with aniline blue solution for 10 min, rinsed in distilled water and differentiated in 1% acetic acid solution for 2 min, washed in water and dehydrated through graded ethyl alcohol before being cleared in xylene and mounted in DPX mounting media. Masson’s-stained skin slides were imaged using the Metafer Vslide Scanner (MetaSystems) with a Zeiss Axio Imager Z2 at 20 × magnification and scored in a blinded manner by two independent investigators as described previously [24]. Skin samples were scored for fibrosis evident by five characteristics (score of 0 to 2 per category): epidermal interface changes, dermal collagen thickness, mononuclear cell inflammation, subdermal fat loss and follicular dropout. Dermal thickness of Masson’s-stained sections was quantified in a blinded manner using FIJI ImageJ software.

### Magnetic resonance imaging (MRI) and diffusion tensor imaging (DTI)

Ex vivo MRI and DTI were performed exactly as described previously [25, 26]. Briefly, brains were collected in-skull, with the ventral side of the cranium opened up to allow diffusion of contrast agent (0.2% gadopentetate dimeglumine; Magnevist®, Bayer Healthcare) over a course of 4 days at 4 °C. Brains were then placed into Fombulin oil (perfuoropolyether solution, Solvay Solexis, NJ) and high-resolution imaging conducted using a Bruker 16.4 T small animal MR imaging system with a vertical wide bore, equipped with a Micro2.5 gradient set and a 15 mm linear saw coil (M2M imaging). High-resolution T1/T2 imaging was performed using Paravision software (version 6.0.1) and a gradient echo fast low-angle shot (FLASH) MRI sequence, with TR/TE = 40/10 ms and a 30-degree flip angle; the acquisition time was 17 min 24 s. Images were acquired using a 20 × 12.7 × 9 mm field-of-view with 400 × 256 × 180 matrix to produce 180 slices with 50 μm slice resolution. DT images were acquired using echo time = 17 ms, repetition time = 100 ms, 20 × 12.8 × 9 mm field of view, 133 × 86 × 60 image size with 0.15 mm slice resolution and the acquisition time was 42 min 32 s. DTI eigenvalues were computed using Paravision (version 6.0.1), and DTI eigenvalues measured using ITK–SNAP software for the following ROIs: corpus callosum, hippocampal commissure, and anterior commissure. T1/T2 FLASH scans were masked using ITK–SNAP software to remove imaged skull bones and then mapped to the Australian Mouse Brain Mapping Consortium (AMBMC) template using FMRIB’s linear and nonlinear image registration tools: FLIRT and FNIRT. ITK–SNAP software was used to measure the volumes of various brain regions (e.g., the hippocampus).

### Immunofluorescence staining

Prior to immunofluorescent staining, sections underwent deparaffinization in xylene, were sequentially rehydrated in 100%, 95%, 70%, and 50% ethanol, respectively, and then placed in tap water until staining. Sections were first blocked for 2 h using 5% bovine serum albumin (BSA) with 0.3% Triton-X-100 in 1xPBS, followed by overnight incubation at 4 °C with primary antibodies: rabbit anti-IBA1 (1:1000; Wako), rat anti-CD68 (1:500; Sigma-Aldrich), rabbit anti-CD3 (1:250; Dako), rat anti-B220 (1:250; Biolegend), rat anti-Ly6B.2 (1:250; Thermofisher), rat anti-MHCII (1:500, Thermofisher) and/or mouse anti-GFAP (1:1000, Millipore), prepared in 3% BSA with 0.1% Triton-X 100 in PBS. Sections were washed the next day with PBS before incubation in secondary antibodies: Alexa Fluor goat anti-rabbit 488 (1:1000), Alexa Fluor goat anti-mouse 488 (1:1000), Alexa Fluor goat anti-rat 568 (1:1000), Alexa Fluor goat anti-rabbit 647 (1:750) and Alexa Fluor goat anti-rat 647 (1:750) diluted in 3% BSA with 0.1% Triton-X 100 in PBS. For B220 staining, sections were incubated with donkey anti-rat biotinylated fragment, followed by ABC complex. DAB was developed according to manufacturer’s instructions (Sigma-Aldrich). All sections were thoroughly washed in PBS and stained with DAPI (1:1000) for 10 min prior to mounting with Vectashield H-100 medium (Vector labs).

### Image analysis and cell quantification

Sections were imaged on a Diskovery spinning disk confocal microscope using the NIS Elements software (version 5.02, Nikon). Representative images were acquired using 20 × or 60x (oil immersion) objective, with a z-stack at 0.9 μm and 0.3 μm intervals, respectively. For IBA1 and CD68, mean fluorescence intensity (MFI) analysis was conducted using Imaris software, with MFI values calculated from average of four consecutive sections per mouse. For cell quantification, four sections per mouse were quantified throughout the length of epidermis and dermis. Counts were performed using StereoInvestigator software (MicroBrightfield Bioscience) using an AxioImager Z2 microscope (Zeiss) and an ORCA-R2 digital charge-coupled device camera (Hamamatsu). The region to be quantified was first contoured using DAPI, and cells were counted live by movement throughout the z-plane. After obtaining the cell counts throughout the contoured length, cell numbers were normalised to the length of the epidermis and the counts from all sections averaged to obtain the mean value. For hippocampus cell counts, two consecutive sections located at Bregma − 1.50 mm and − 1.70 mm were counted live for each mouse using StereoInvestigator software as mentioned above. Cell counts were normalised to the area of the hippocampus, and the average of two sections recorded.

### Statistical analysis

Statistical analysis was performed using GraphPad Prism software (Version 9.1). Data were analysed using an unpaired two-tailed Student's *t* test, a repeated-measures one-way and two-way ANOVA with Bonferroni post-hoc test, as appropriate. Values represent group mean ± SEM unless specified otherwise, with significance determined at *P < 0.05; **P < 0.01; ***P < 0.001; ****P < 0.0001.

## Results

### PLX3397 treatment improves clinical and histopathological symptoms of cGVHD

**Fig. 1:**
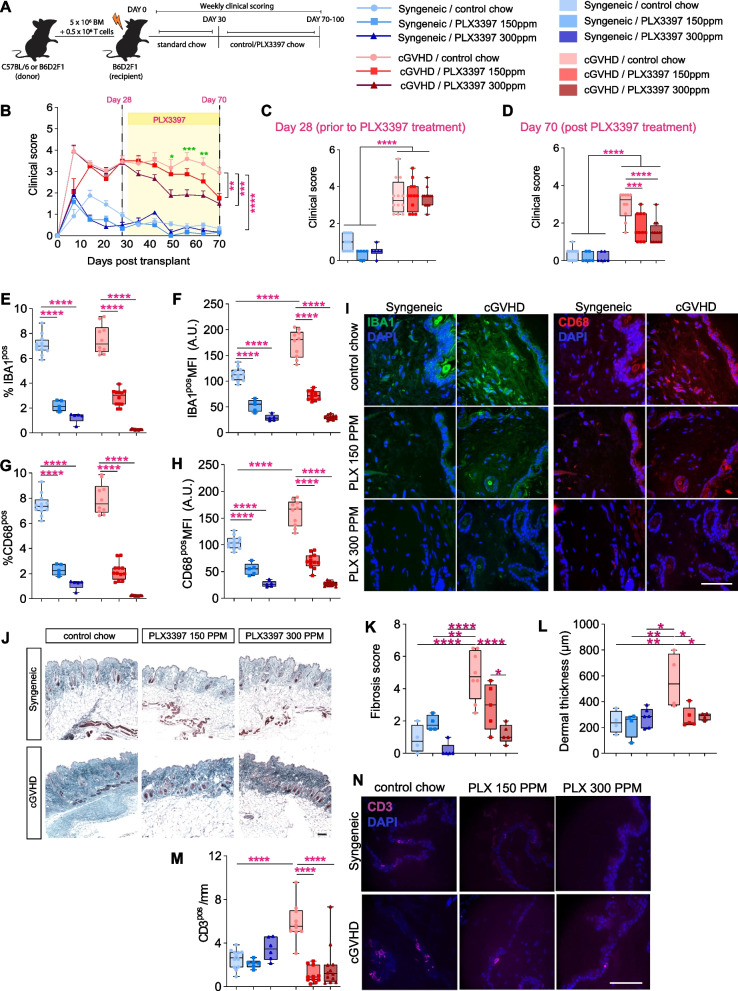
PLX3397 treatment during cGVHD progression attenuates clinical and histopathological outcomes. **A** Experimental timeline for irradiation, cell transplantation and subsequent PLX3397 treatment. Lethally irradiated B6D2F1 mice were transplanted with 5 × 10^6^ bone-marrow cells and 0.5 × 10^6^ mature splenic T cells from either B6D2F1 (syngeneic controls) or C57Bl/6 (allogeneic) donor mice to induce cGVHD. From day 30 post-transplant, mice either continued to receive standard control chow, or were switched to ‘treatment chow’ that incorporated 150 ppm or 300 ppm PLX3397. All mice were scored for pathology weekly until the end of experiment (*n* = 6–12 mice/group). **B** Temporal overview of clinical scores for all experimental mice until sacrifice. cGVHD mice scored higher than syngeneic controls throughout the course of the experiment. PLX3397 treatment at 150 ppm or 300 ppm reduced clinical scores in the cGVHD group. Green asterisks are cGVHD ‘control chow’ versus cGVHD PLX3397 300 ppm treatment on days 56, 63 and 70 post-transplant. Magenta asterisks are cGVHD ‘control chow’ versus cGVHD PLX3397 150 ppm and 300 ppm, and versus ‘syngeneic control chow’ treatment on day 70 post-transplant, respectively. **C** Clinical scores in syngeneic and cGVHD (allotransplant) mice on day 28 post-transplant, prior to the commencement of PLX3397 treatment. Asterisks indicate post-hoc results. **D** Clinical scores in syngeneic mice and cGVHD mice on day 70 post-transplant, after 40 days of control or PLX3397 treatment at 150 ppm or 300 ppm. Note that PLX3397 treatment at either concentration significantly reduced clinical pathology scores in the cGVHD group. Asterisks indicate post-hoc results. **E** Quantification of IBA1^pos^ immunoreactivity (proportional area) in the skin of syngeneic and cGVHD mice, with and without PLX3397 treatment. Asterisks indicate post-hoc results. **F** Quantification of the mean fluorescence intensity (MFI) of IBA1^pos^ cells in the skin of syngeneic and cGVHD mice, with and without PLX3397 treatment. Values shown are in arbitrary units (A.U.). Asterisks indicate post-hoc results. **G**, **H** Quantification of CD68^pos^ immunoreactive area (**G**) and CD68^pos^ MFI (**I**) in skin tissue from syngeneic and cGVHD mice, with and without PLX3397 treatment. Asterisks indicate post-hoc results. **I** Representative confocal images of IBA1^pos^ (green) and CD68^pos^ (red) macrophages in the skin of syngeneic and cGVHD mice, with and without PLX3397 treatment. DAPI (blue) was used to label cell nuclei. **J** Masson’s trichrome stained skin samples of syngeneic and cGVHD treated with control or PLX3397 chow. **K**, **L** Fibrosis score (**K**) and dermal thickness (**L**) of skin tissue stained with Masson’s trichrome staining from syngeneic and cGVHD mice with and without PLX3397. **M** Quantification of CD3^pos^ T-cell numbers in the epidermal layers of the skin from syngeneic and cGVHD mice, with and without PLX3397 treatment. Note that cGVHD mice had an increased density of CD3^pos^ T cells compared to syngeneic controls, an effect that was ameliorated by PLX3397 treatment. Asterisks indicate post-hoc results. **N** Representative confocal images of CD3^pos^ T cells from syngeneic and cGVHD mice, with and without PLX3397 treatment. Statistics: repeated measure two-way ANOVA with Bonferroni’s multiple comparisons (**B**) one-way ANOVA (**C–H**, **K–M**) followed by Bonferroni post hoc comparison. Dots in box plots represent individual mice. *p < 0.05, **p < 0.01, ***p < 0.001, ****p < 0.0001. Scale bar = 50 µm (**I**, **N**), 100 µm (**J**)

To assess the putative efficacy of PLX3397 as a therapeutic intervention for cGVHD, we first induced this disease using the established ‘C57BL/6 into B6D2F1’ model of sclerodermatous cGVHD, in which a low dose of T cells induces cGVHD pathology from day 30 onwards [18–20, 27, 28] (Fig. 1A). This mouse model successfully recapitulates multi-organ pathology as evident clinically, thereby making this model reliable to study cGVHD pathophysiology [20]. Moreover, due to being a parent to F1 model, C57BL/6 [H2^b^] → ; B6D2F1 [H2^b/d^] representing haploidentical immunologic disparity, this model is more reflective of clinical situation, where haploidentical hematopoietic transplants are more common than exact MHC matched transplants. Briefly, lethally irradiated B6D2F1 mice were transplanted with either syngeneic (B6D2F1) or allogeneic (C57BL/6) bone marrow. In allotransplant recipients, the bone-marrow mixture was supplemented with 0.5 × 10^6^ mature splenic T cells to induce cGVHD. All transplanted mice were kept on standard chow until 30-day post-transplantation, before being divided into different PLX3397 treatment groups. Control cGVHD mice continued standard chow until sacrifice post day 70, while treatment groups received chow incorporated with either 150 ppm or 300 ppm of PLX3397 from day 30 onwards (i.e., when cGVHD is established) to deplete microglia/macrophages.

To confirm the onset and progression of cGVHD, mice were scored weekly by examining their weight, skin texture, fur integrity, movement and posture [21], with a higher clinical score reflecting a worse disease phenotype (Table 1). Throughout the experiment, cGVHD mice consistently had higher clinical scores compared to syngeneic control mice (Fig. 1B). At 28-day post-transplant (i.e., just prior to the commencement of PLX3397 treatment), there were no significant differences in clinical scores between any cGVHD mice (Fig. 1B, C). However, from post-transplant day 49 onwards (i.e., after 19 days of PLX3397 treatment), cGVHD mice treated with 300 ppm PLX3397 displayed significantly lower clinical scores compared to cGVHD control mice (Fig. 1B). Although both concentrations of PLX3397 resulted in similar improvements in clinical scores in cGVHD mice by post-transplant day 70, the 300 ppm PLX3397 group displayed clinical improvements sooner than the 150 ppm treatment group (Fig. 1B, D). PLX3397 treatment did not affect the clinical scores of syngeneic mice. Taken together, these results demonstrate that PLX3397 treatment improves the clinical outcomes of cGVHD. Although both concentrations had similar efficacy by post-transplant day 70, the earlier emergence of clinical improvements in the 300 ppm treatment group shows that the observed benefits of PLX3397 treatment occur in a dose-dependent manner.

As skin is significantly affected by cGVHD [29], we next assessed the peripheral effects of PLX3397 treatment on cGVHD-associated skin pathology. For this, we first confirmed that PLX3397 treatment resulted in macrophage depletion from the skin at 70-day post-transplant and 40 days after the initiation of PLX3397 treatment. Skin sections were stained for the pan-macrophage markers, ionized calcium-binding adaptor molecule 1 (IBA1) [30] and Cluster of Differentiation 68 (CD68) [31]. In animals fed with control chow, the immunoreactive area for IBA1- and CD68-positive cells were not significantly different between the syngeneic control and cGVHD groups (Fig. 1E, G). However, the mean fluorescence intensity (MFI) for both these stains were significantly higher in the cGVHD control group compared to the syngeneic group (Fig. 1F, H, I), which confirms that cGVHD induces an activated macrophage phenotype [30]. As expected, PLX3397 treatment at both 150 ppm and 300 ppm significantly reduced the percentage of IBA1- and CD68-positive cells (in a dose-dependent manner) in both syngeneic and cGVHD mice (*p* < 0.0001; Fig. 1E, G). Consistent with the loss of these cells, IBA1 and CD68 MFI values were also significantly reduced in both syngeneic and cGVHD mice (Fig. 1F, H). Taken together, these findings confirm that PLX3397 treatment can induce a near-complete depletion of macrophages from the skin of syngeneic and cGVHD mice (Fig. 1E–I). We additionally confirmed that PLX3397 treatment resulted in IBA1^pos^ macrophage depletion in liver samples compared with control chow mice (Additional file 1: Fig. S1A–C).

After confirming skin macrophage depletion by PLX3397, we next investigated whether the treatment had any effect on cGVHD-induced skin pathology. A defining characteristic of cGVHD is scleroderma and fibrosis of the skin, and here we confirmed that these characteristics were observed in our cGVHD group using Masson’s trichrome staining of skin samples (Fig. 1J–L). Skin fibrosis was assessed in a blinded manner defined by epidermal interface changes, dermal collagen thickness, mononuclear cell inflammation, subdermal fat loss and follicular dropout, as described previously [24]. cGVHD control chow mice exhibited significantly greater skin fibrosis scores compared to syngeneic controls and PLX3397 treatment significantly reduced skin fibrosis of cGVHD mice (Fig. 1K). In addition, fibrosis was evident by the increased thickness of the skin dermis in cGVHD control mice compared to syngeneic controls and this was reduced by PLX3397 treatment (Fig. 1L). In addition, the cGVHD group that was fed normal chow had markedly higher histopathology scores in analysed skin tissues compared to syngeneic control mice (Additional file 1: Fig. S1D), as evident by a more widespread separation between the dermal and epidermal layers (Additional file 1: Fig. S1E, arrowheads), along with infiltration of lymphocytes (Additional file 1: Fig. S1D, E). Notably, cGVHD mice maintained on PLX3397-containing chow, at either 150 ppm or 300 ppm, had significantly lower skin pathology scores compared with cGVHD control mice (Additional file 1: Fig. S1D, E). In addition to improved outcomes in the skin, PLX3397-treated mice also displayed reduced mononuclear cell infiltration, improved bronchiolar structural integrity, and reduced mucosal denudation in the liver, lung, and the gut tissues, respectively (Additional file 1: Fig. S1F–K)*.* Notably, while high-dose PLX3397 treatment at 300 ppm resulted in lower histopathology scores in all four tissues examined, lower dose treatment at 150 ppm only improved histopathology in the gut and skin (Additional file 1: Fig. S1D, E, J, K)*.*

Given that PLX3397 treatment reduced skin histopathology in cGVHD mice, we next investigated the impact of macrophage depletion on the immune landscape within the skin tissue. In line with previous findings [32], we found a significant increase in the number of CD3^pos^ T cells in the cGVHD control group compared to the syngeneic control group (Fig. 1M, N). Strikingly, PLX3397 treatment significantly attenuated the cGVHD-induced abundance of CD3^pos^ T cells in skin tissue, and to a comparable degree (Fig. 1M). In contrast, B220^pos^ B-cell numbers (which were also increased as a result of cGVHD compared to syngeneic controls) were unaffected by the PLX3397 treatment (Additional file 1: Fig. S1L). The density of Ly6B.2^pos^ neutrophils in the skin was similarly not different between any of the groups investigated (Additional file 1: Fig. S1M)*.* Taken together, these results indicate that PLX3397 treatment specifically influences the abundance of T cells within the altered immune landscape of the skin.

### PLX3397 treatment prior to the onset of cGVHD does not improve disease outcomes

**Fig. 2:**
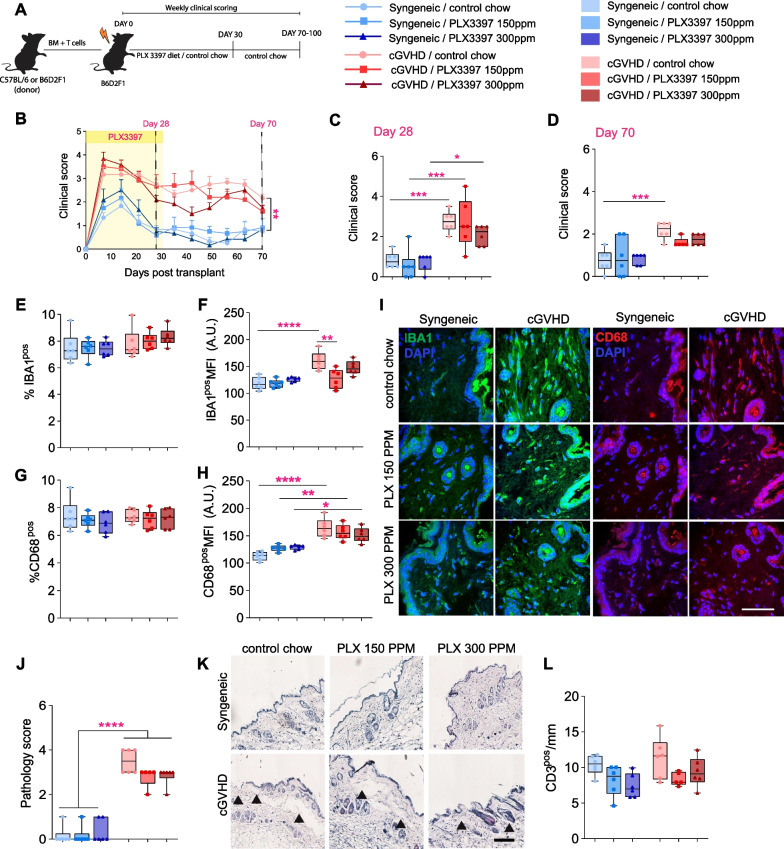
PLX3397 pre-treatment (days 0–30 post-transplant) does not prevent the onset nor attenuate clinical symptoms and histopathology of cGVHD. **A** Experimental timeline for irradiation, adoptive cell transfer and PLX3397 treatment. Lethally irradiated B6D2F1 mice were transplanted with 5 × 10^6^ BM cells and 0.5 × 10^6^ mature splenic T cells from either B6D2F1 (syngeneic controls) or C57Bl/6 (allogeneic) donor mice to induce cGVHD. From post-transplant days 0–30, mice were also fed either control chow or PLX3397-incorporated chow at 150 ppm or 300 ppm, before being all switched back to standard chow for post-transplant days 30–70. Mice were scored for pathology weekly until post-transplant day 70 (*n* = 5–6 mice/group). **B** Temporal profile of clinical scores over the duration of the experiment, i.e., until day 70 post-transplant. Note that cGVHD mice consistently scored higher than syngeneic controls. PLX3397 pre-treatment did not reduce clinical scores, either during the treatment period (post-transplant days 0–30) or during the 40 days of cGVHD progression (post-transplant days 30–70). Magenta asterisks are ‘cGVHD/control chow’ versus ‘Syngeneic/control chow’. **C** Clinical scores in syngeneic and cGVHD mice on day 28 post-transplant, control chow or PLX3397 treatment at 150 ppm or 300 ppm. Note that PLX3397 pre-treatment (i.e., prior to cGVHD development) had no impact on clinical symptoms. Asterisks indicate post-hoc results. **D** Clinical scores in syngeneic and cGVHD mice on day 70 post-transplant, with 30 days of control or PLX3397 pre-treatment (at 150 ppm or 300 ppm) followed by 40 days of control chow. Note that PLX3397 pre-treatment had no impact on the severity of the clinical symptoms of cGVHD. Asterisks indicate post-hoc results. **E**, **F** Quantification of the IBA1^pos^ immunoreactive area (**E**) and IBA1^pos^ mean fluorescence intensity (MFI) values (F) in skin tissue from syngeneic and cGVHD mice, with and without PLX3397 pre-treatment. Asterisks indicate post-hoc results. **G** (Left) Representative confocal images of IBA1^pos^ macrophages (green) in the skin. Cell nuclei are labelled with DAPI (blue). (Right) Representative confocal images of CD68^pos^ macrophages (red) in the skin from syngeneic and cGVHD mice, with and without PLX3397 pre-treatment. Cell nuclei are labelled with DAPI [blue (**H**, **I**) quantification of the CD68^pos^ immunoreactive area (**H**) and CD68 MFI values (**I**)] in skin tissue from syngeneic and cGVHD mice, with and without PLX3397 pre-treatment. Asterisks indicate post-hoc results. **J**, **K** Histopathology scores (**J**) and representative images of skin tissue stained with haematoxylin and eosin (**K**) from syngeneic and cGVHD mice, with and without PLX3397 pre-treatment. Arrowheads point at cGVHD pathology in the skin as per Table 2. **L** Mansson’s trichrome stained skin samples from syngeneic and cGVDH treated with control or PLX3397 chow. Scale bar = 100 µm. **M** Fibrosis score for skin samples from syngeneic and cGVHD mice, with and without PLX3397 pre-treatment. Asterisks indicate post-hoc results. **N** Dermal thickness quantified using Masson’s trichrome stained skin samples. Asterisks indicate post-hoc results. **O** Quantification of CD3^pos^ T cells in the epidermal layer of skin tissue from syngeneic and cGVHD mice, with and without PLX3397 pre-treatment. Asterisks indicate post-hoc results. **P** Representative confocal images of CD3^pos^ T cells in the skin from syngeneic and cGVHD mice, with and without PLX3397 pre-treatment. Statistics: repeated measure two-way ANOVA with Bonferroni’s multiple comparisons (**B**), two-way ANOVA (**C–H**, **J**, **M–O**) followed by Bonferroni post hoc comparison. Dots in box plots represent individual mice. *p < 0.05, **p < 0.01, ***p < 0.001, ****p < 0.0001. Scale bar = 100 µm (**L**, **P**), 50 µm (**G**, **J**)

The above results showed that PLX3397 treatment can significantly attenuate cGVHD-induced histopathology when introduced at disease onset (from day 30 post-transplant). However, this treatment regime did not completely revert clinical scores to those of syngeneic controls (Fig. 1B, D). Therefore, we next tested whether commencing PLX3397 treatment immediately after transplantation could prevent the onset and/or development of cGVHD altogether. To test this, B6D2F1 recipients were lethally irradiated and placed on either control chow or PLX3397-incorporated chow (at 150 ppm or 300 ppm) immediately after transplantation. After post-transplant day 30, all mice were switched onto control chow until day 70; clinical and histopathological analyses were performed as described above (Fig. 2A). We found once more that cGVHD mice maintained higher clinical scores than syngeneic mice throughout the course of the experiment, but in contrast to when treatment was initiated at cGVHD onset, PLX3397 pre-treatment did not prevent or attenuate GVHD symptoms over the 30-day post-transplant (Fig. 2B, C). Furthermore, no significant differences were observed between cGVHD groups at post-transplant day 70 (Fig. 2D). These results demonstrate that pre-treatment with PLX3397 does not prevent the onset or severity of the clinical symptoms of cGVHD**.**

To assess the long-term effect of PLX3397 pre-treatment on macrophage repopulation and/or presence at 70-day post-transplant, we again performed immunostaining for IBA1 and CD68. As specified above, syngeneic and cGVHD mice were fed control chow or PLX3397-incorporated chow for the first 30 days after transplant, following which all mice were placed on control chow until 70-day post-transplant for post-mortem histological analyses. We observed no differences in the IBA1- and CD68-positive immunoreactive area between syngeneic and cGVHD mice treated with control chow (Fig. 2E, G, H, I, respectively), but did again detect an elevated MFI for both IBA1 and CD68 in the cGVHD group, which agrees with our earlier observations. (Fig. 2F–I). However, in contrast to our findings above, PLX3397 treatment prior to the onset of cGVHD did not attenuate any of these changes, except in the ‘cGVHD/PLX3397 150 ppm’ group in which the IBA1 MFI was significantly reduced compared the ‘cGVHD/control chow’ group (Fig. 2E–I). Taken together, we conclude that the skin macrophage population in mice pre-treated with PLX3397 recovers to control levels by 70-day post-transplant.

We then investigated the impact of PLX3397 pre-treatment on cGVHD-induced histopathology and the composition of the peripheral immune infiltration into the skin more broadly. As expected, the cGVHD group fed normal chow displayed higher skin pathology scores compared to syngeneic control mice (Fig. 2J, K). However, consistent with clinical observations, there was no significant reduction in histopathology with PLX3397 pre-treatment (at either 150 ppm or 300 ppm), as evident by the persistent presence of dermal and epidermal separation in these mice (Fig. 2J, K). Likewise, none of the other cGVHD target organs investigated (lungs, liver, gut) displayed any reductions in histopathology with PLX3397 pre-treatment (Additional file 2: Fig. S2A–G)*.* Finally, unlike our findings reported above (Fig. 1O), CD3^pos^ T cells were also not significantly changed in this experimental paradigm (Fig. 2L). B cell and neutrophil presence was also not impacted by PLX3397 pre-treatment (data not shown).

Taken together, our results demonstrate that pre-treatment with PLX3397 neither prevents the onset of cGVHD, nor attenuates the severity of clinical and histopathological cGVHD symptoms. Thus, to confer therapeutic benefits in cGVHD, PLX3397 treatment must be applied in a timing-dependent manner that coincides with the onset of disease symptoms.

### Therapeutic PLX3397 treatment improves cGVHD-associated memory impairments and reduces neuroinflammation

**Fig. 3:**
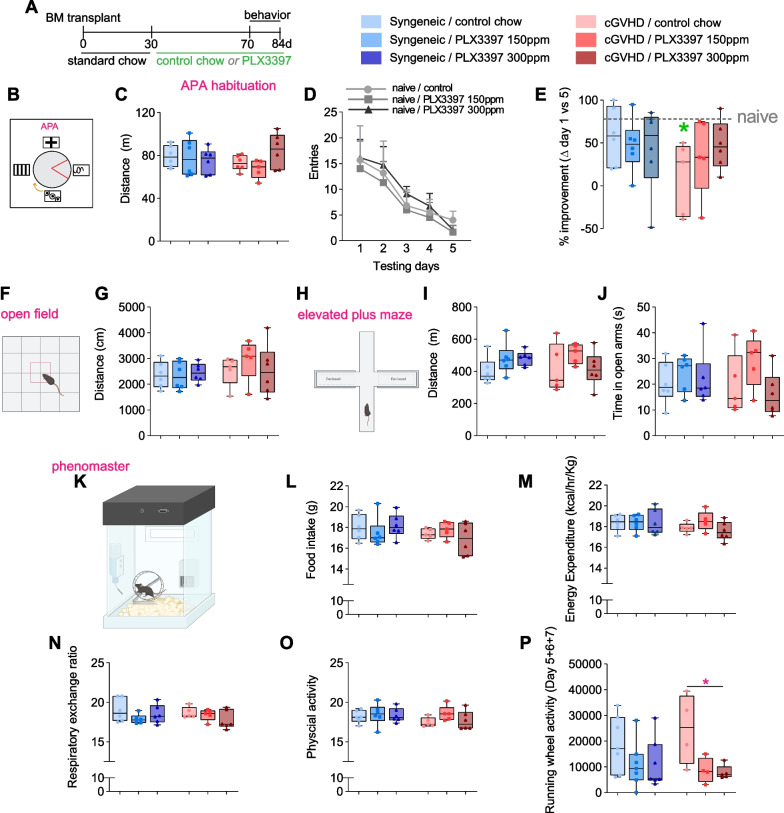
Therapeutic PLX3397 treatment (days 30–70 post-transplant) attenuates cGVHD-associated memory impairment. **A** Experimental timeline for bone-marrow transplantation, administration of either control or PLX3997-containing chow, and behavioural testing from day 70 post-transplant. **B** Schematic of visual cues and shock zone location used in the active place avoidance (APA) task. **C** Distance travelled in the APA paradigm on the day 0 (habituation day, shock zone off) between syngeneic mice and cGVHD mice, with and without PLX3397 treatment. **D** Entries into the shock zone for naïve mice showing spatial learning over a course of five testing days, which is unchanged by PLX3397 treatment compared with control chow. **E** Percentage improvement in APA performance on day 5 versus day 1 between syngeneic mice and cGVHD mice, with and without PLX3397 treatment. Note cGVHD mice on control chow had a significantly worse performance compared with naïve controls (dotted line, green asterisk), while the cGVHD treated with PLX3397 (150 ppm or 300 ppm) had a comparable APA performance compared to their syngeneic counterparts and naïve controls. **F** Schematic of open field task. **G** Distance travelled during the open field task. **H** Schematic of the elevated plus maze task. **I** Distance moved during the elevated plus maze task. **J** Time spent in the open arms during the elevated plus maze task. **K** Schematic of the phenomaster. Mice are provided a running wheel and food and water ad libitum. Various locomotion and metabolic measures are tracked by automatic sensors (represented by the black box). **L** Food intake measurement between syngeneic and cGVHD mice, with and without PLX3397 treatment. **M** Energy expenditure measurement between syngeneic and cGVHD mice, with and without PLX3397 treatment. **N** Respiratory exchange ratio measurement between syngeneic and cGVHD mice, with and without PLX3397 treatment. **O** Physical activity measurement between syngeneic and cGVHD mice, with and without PLX3397 treatment. P Running wheel activity of individual mice on days 5, 6 and 7 of the week in which they were given access to a running wheel. Statistics: one-way AONVA followed by Bonferroni post hoc comparisons (**C**, **E**, **G**, **I**, **J**, **L–P**), or two-way ANOVA with repeated measures and Bonferroni post hoc comparisons (**D**). APA: active place avoidance. *p < 0.05, **p < 0.01, ***p < 0.001, ****p < 0.0001

We recently identified the brain as a target organ that is adversely affected by cGVHD, evidenced by neuroinflammation and cognitive deficits [13]. PLX3397 readily crosses the BBB, thus we hypothesised that PLX3397 treatment may attenuate the behavioural and neuroinflammatory impacts of cGVHD. To test this hypothesis, mice from our first series of experiments (treated with control chow or PLX3397 from post-transplant day 30) were additionally tested in a variety of behavioural tasks from post-transplant day 70 onwards (Fig. 3A). To assess higher cognitive functioning, mice were subjected to the active place avoidance (APA) task to test for hippocampal-dependent spatial learning and memory (Fig. 3B). The APA test requires that mice use visual cues on the walls of the testing room to actively avoid a stationary shock zone, located in one of the quadrants of a rotating circular arena [22, 33]. At baseline, mice from all groups exhibited similar locomotive behaviour with no differences in total distance travelled (Fig. 3C). Naïve mice quickly learned to actively avoid the shock zone across 5 days of APA testing, reducing their shock zone entries over time; PLX3397 treatment did not alter the learning of naïve mice (Fig. 3D). Similarly, mice in the syngeneic group were also able to learn the APA task (as evident from the percentage improvement, which reflects the significant reduction in the number of shock zone entries on day 5 of testing compared to day 1; Fig. 3E); their ability to successfully acquire this task was again irrespective of whether they were fed control or PLX3397-containing chow. cGVHD mice performed significantly worse than syngeneic mice, with little to no improvement in APA task performance over the testing period, and their learning and memory abilities were significantly below those of naïve controls (Fig. 3E). Importantly, PLX3397 treatment substantially improved in the learning ability of cGVHD mice in the APA task, with their performance being comparable to syngeneic controls and also not significantly different from naïve controls (Fig. 3E). Together, these results suggest that PLX3397 treatment can, at least in part, attenuate the cognitive deficits of cGVHD.

Beyond APA learning, we also assessed general locomotor abilities of mice in the open field task, where mice were allowed to freely explore the testing arena. We found no difference between syngeneic and cGVHD mice, with or without PLX3397 treatment (Fig. 3F,G). Anxiety-like behavior of mice was assessed in the elevated plus maze task, involving open and closed arms (Fig. 3H); mice were typically averse to the open arms (data not shown), and there was no difference in the total distance covered (Fig. 3I), or the time spent in the open arms (Fig. 3J), between syngeneic and cGVHD groups with and without PLX3397 treatment.

It is known that cGVHD impacts metabolic function [34], and we, therefore, we assessed whether the depletion of microglia/macrophages altered the metabolic profile during cGVHD by subjecting these mice to PhenoMaster assessment. The PhenoMaster system is a non-invasive automated physiological measuring system that records various metabolic activities of animals (e.g., oxygen consumption, energy expenditure, food intake etc.), along with their physical activity (Fig. 3K). We did not find any significant differences in food intake, energy expenditure, physical activity, or the respiratory exchange ratio (RER) between any of the cGVHD mice and syngeneic transplant groups (Fig. 3L–O), suggesting PLX3397 incorporation did not alter eating habits of animals or their general well-being. Mice also had access to a freely moving running wheel in their PhenoMaster cages. As they were individually housed, we could assess the individual running wheel activity for each mouse. We found that cGVHD mice on control chow appeared to have greater running wheel activity compared to those receiving 300 ppm PLX3397 (Fig. 3P). Overall, these data show that there were minimal and/or no changes in the various physiological parameters measured by the PhenoMaster system during cGVHD progression, and also that these were not overtly influenced by PLX3397 treatment in our experimental paradigm.

**Fig. 4:**
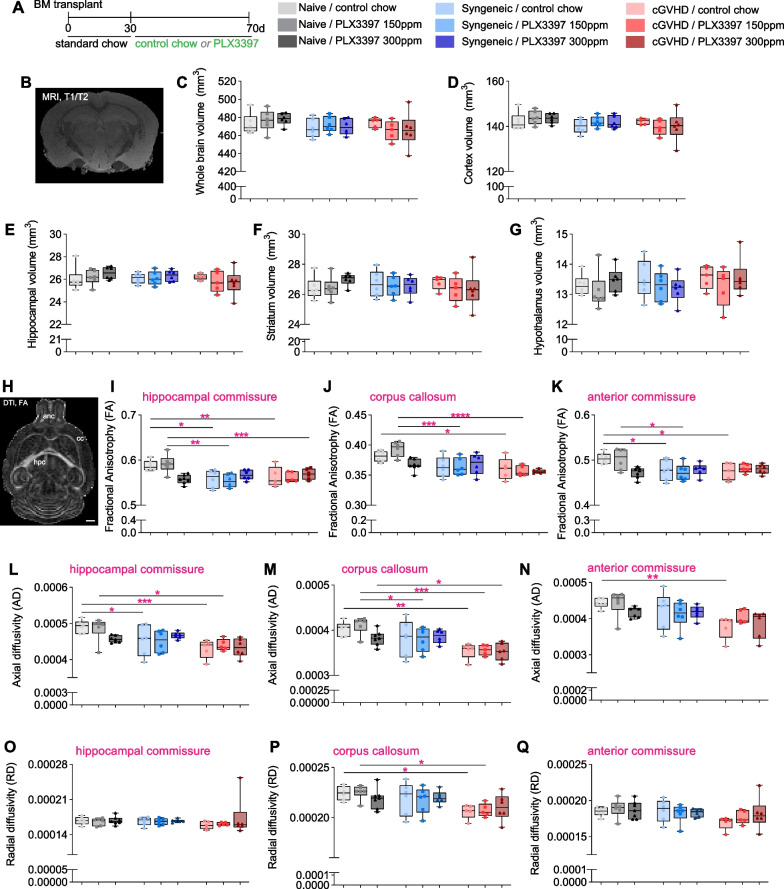
Brain volume and diffusion tensor imaging (DTI) metrics in cGVHD mice and syngeneic transplant controls, with and without PLX3397 treatment. **A** Experimental timeline for irradiation, transplant and subsequent PLX3397 treatment. **B**, **C** Representative coronal image of ex vivo MRI T1/T2 flash image (**B**) and hippocampal volume (**C**). **D** Ex vivo sagittal image of fractional anisotropy (FA) displaying the hippocampal commissure (hpc), corpus callosum (cc) and anterior commissure anc). **E–G** Fractional anisotropy (FA) of the hippocampal commissure (**E**), corpus callosum (**F**), and anterior commissure (**G**). **H–J** Axial diffusivity (AD) of the hippocampal commissure (**H**), corpus callosum (**I**), and anterior commissure (**J**). **K–M** Radial diffusivity of the hippocampal commissure (**K**), corpus callosum (**L**), and anterior commissure (**M**). Dots represent individual mice. **B**, **D** Scale bars: 1 mm. Statistics: two-way ANOVA with Bonferroni post-hoc comparisons (**C**, **E–M**). *p < 0.05, **p < 0.01, ***p < 0.001, ****p < 0.0001

We next assessed whether the observed behavioural changes in APA acquisition by cGVHD mice were associated with any gross changes in hippocampal volume or brain connectivity (Fig. 4A, B). For this, we conducted high-resolution magnetic resonance imaging (MRI) and diffusion tensor imaging (DTI), respectively, using a small animal 16.4 T Bruker imaging system. To account for any changes in outcomes being due to irradiation and/or BM transplantation rather than cGVHD, we also included a cohort of naïve mice here. We found no significant difference in the volume of any brain region examined between experimental groups (Fig. 4C–G). Diffusion tensor measures did appear to show a reduction in fractional anisotropy (FA) values for three of brain’s major tracts, namely, the hippocampal commissure, corpus callosum and the anterior commissure in cGVHD mice (Fig. 4H–K). However, these changes in FA values were not significantly different from syngeneic transplant controls, indicating an influence of the irradiation and transplant procedure itself rather than and/or in addition to cGVHD. Changes in FA across the three fibre tracts appeared to be mostly driven by reduced axial diffusivity values (which represent a surrogate biomarker of axonal integrity[25, 35]), and most profoundly so in cGVHD mice (Fig. 4L–N). Only for the corpus callosum did we observe additional changes in radial diffusivity, a measure affected by both myelination status and gliosis [25, 35]; these changes were only present in cGVHD mice and not syngeneic transplant controls (Fig. 4O–Q). None of the above detailed readouts and/or changes were affected by PLX3397 treatment.

**Fig. 5:**
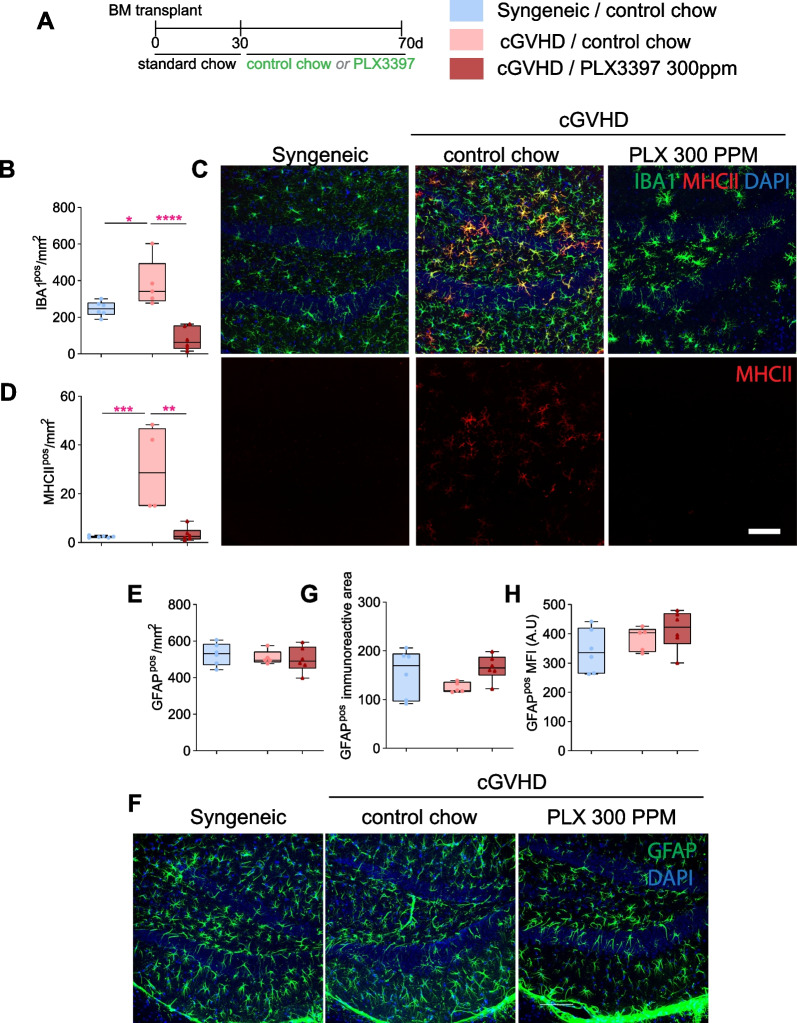
Therapeutic PLX3397 treatment (days 30–70 post-transplant) reduces neuroinflammation in the cGVHD brain. **A** Experimental timeline for control chow and/or PLX3397 (300 ppm) treatment, given between days 30 and 70 post-transplant, for syngeneic and cGVHD mice. **B** Quantification of IBA1^pos^ cell density in the hippocampus of syngeneic controls and cGVHD mice with and without PLX3397 treatment (days 70–100 post-transplant). Asterisks indicate post-hoc results. **C** Representative confocal images of IBA1^pos^ (green) and MHCII^pos^ (red) cells in the hippocampus of syngeneic controls and cGVHD mice with and without PLX3397 treatment Scale bar = 100 µm. **D** Quantification of MHCII^pos^ cell density in the hippocampus of syngeneic controls and cGVHD mice with and without PLX3397 treatment (days 70–100 post-transplant). Asterisks indicate post-hoc results. **E** Quantification of GFAP^pos^ cell density in the hippocampusof syngeneic controls and cGVHD mice with and without PLX3397 treatment (days 70–100 post-transplant). **F** Representative confocal images of GFAP^pos^ cells (green) in the hippocampus of syngeneic controls and cGVHD mice with either control chow or PLX3397 (300 ppm) treatment. Cell nuclei are labelled with DAPI (blue). Scale bar = 100 µm. **G** Quantification of the GFAP immunoreactive area in the hippocampus of syngeneic controls and cGVHD mice with and without PLX3397 treatment (**H**) Quantification of the mean fluorescence intensity (MFI) for GFAP staining in the hippocampus of syngeneic controls and cGVHD mice with and without PLX3397 treatment. Statistics: one-way ANOVA (**B**, **D**, **E**, **G**, **H**) followed by Bonferroni post hoc comparison. **C**, **F** Scale bar: 100 µm. Dots in box plots represent individual mice. *p < 0.05, **p < 0.01, ***p < 0.001, ****p < 0.0001

We finally assessed the neuroinflammatory microenvironment in the hippocampus of cGVHD mice with and without PLX3397 treatment, and syngeneic controls (Fig. 5A). In the healthy brain, MHC II expression on microglia (which plays an integral role in antigen presentation to T cells) rarely occurs. In sharp contrast, MHC II is prominently expressed by microglia as well as donor-derived peripheral macrophages that infiltrate the brain during cGVHD [13]. Our previous report suggested a critical involvement of MHC II in driving brain cGVHD pathology, as allogeneic bone-marrow grafts from MHC II knockouts resulted in attenuated neuroinflammation and behavioural improvements [13]. Here, we tested whether PLX3397 treatment could counter cGVHD-induced MHC II expression and/or removed these cells from the cGVHD brain. In agreement with our previous study [13], IBA1 staining showed an increase in the overall myeloid cell density in the hippocampus of cGVHD control mice compared to those receiving syngeneic transplants (Fig. 5B, C). In addition, IBA1^pos^ cells in cGVHD control mice displayed increased MHCII reactivity compared to their syngeneic counterparts, as expected (Fig. 5C, D). PLX3397 treatment dramatically reduced both the density and MHCII reactivity on myeloid cells by 79% and 89%, respectively, compared to cGVHD controls (Fig. 5C, D). Glial fibrillary acidic protein (GFAP) immunoreactivity and density of reactive astrocytes, another hallmark of neuroinflammation[36], showed no significant differences between the syngeneic, cGVHD control and PLX3397-treated groups (Fig. 5E–H). Overall, these results demonstrate that PLX3397 treatment depletes microglia–macrophages and/or abolishes MHC-II expression on these cells in the cGVHD hippocampus without any concomitant effect on astrocytes.

## Discussion

Chronic GVHD is a complex syndrome that manifests as a heterogeneous disease. Presently, steroid-based immunosuppressive therapies are the first line of defence against cGVHD; however, their use remains limited due to nonspecific immune suppression, drug toxicity, and the requirement for long-term treatment [37]. Although treatment options are available to counteract these drawbacks, not all patients respond to these [38], highlighting the need for new and more specific approaches to inhibit cGVHD progression. Donor bone-marrow-derived macrophages and host microglia are thought to contribute to the pathogenesis of cGVHD [13]. In the present study, we depleted both these cells using the blood–brain barrier permeable CSF-1R-inhibitor Pexidartinib (PLX3397) to directly test this premise. In doing so, we reveal the therapeutic impacts of this drug on both peripheral- and CNS-specific outcomes in cGVHD. Using varying doses of PLX3397, our data suggest that the microglia/macrophages depletion efficiency is an important consideration as this had dose-dependent effects on cGVHD pathology. While no mouse models to date can recapitulate all the clinical features of cGVHD, our results highlight PLX3397 as a potential new treatment option in those forms of cGVHD, where macrophages are critically involved and/or the dominant cell type driving the onset and persistence of cGVHD.

### PLX3397 treatment during cGVHD development improves clinical and histopathological outcomes

Regardless of the acute or chronic form of this disease, macrophage infiltration is considered a clinical biomarker for GVHD occurrence and persistence [39]. High macrophage infiltration rates are also positively correlated with GVHD severity [40]. Macrophages release TGFβ upon sequestration in the skin, which in turn drives fibrosis by activating fibroblasts and triggering collagen production [7, 27]. Here, we used PLX3397 to deplete macrophages and microglia, subsequently examining the impact of this treatment on clinical outcomes and cGVHD pathology. Of note, PLX3397 treatment for CSF-1R inhibition acts by depleting microglia/macrophages and does not impact myelopoiesis [41]. We observed significantly improved clinical scores in PLX3397-treated cGVHD mice compared to non-treated controls when PLX3397 treatment coincided with disease onset and persistence (i.e., days 30–70 post-transplant). Of note, while the clinical scoring system employed in the present study, first introduced by Cooke et al., 1996 reflects the overall well-being of the mouse, in the context of cGVHD, it is important to have a scoring system that not only assesses the overall condition of the mice but also quantifies specific changes related to the skin, such as alopecia, scaling and skin thickening [42]. We additionally confirmed skin scleroderma and fibrosis was evident in our cGVHD mice using Masson’s trichrome staining. For clinical scoring, mice that received a higher dose of this drug (300 ppm) exhibited improvements sooner than receiving a lower concentration (150 ppm), but their clinical scores were ultimately comparable at the experimental endpoint. Post-mortem studies confirmed that PLX3397 treatment elicited a profound depletion of macrophages in the skin, which coincided with significantly reduced cutaneous pathology, as evident from the preservation of dermal and epidermal layers of the skin and reduced skin fibrosis scores and dermal thickness, as well as diminished CD3^pos^ lymphocytic infiltrate. PLX3397 treatment also reduced histopathology scores for the gut, liver, and lungs in a dose-dependent manner. CSF-1R inhibition does not directly induce T-cell depletion [43] and CSF-1R protein is not expressed by hematopoietic stem cells [44]. Hence, we suggest that PLX3397 effects on reducing T-cell presence in cGVHD affected tissues (e.g., skin and brain) are because of microglia/macrophage depletion. In other words, microglia/macrophages appear to mediate the infiltration and/or expansion of the T-cell pools in cGVHD affected organs. Taken together, these results show that PLX3397 is an effective therapy to treat the pathological manifestations of cGVHD in multiple target organs.

Despite the effective depletion of skin macrophages and marked improvements in histopathological and clinical cGVHD symptoms, it is worth noting that PLX3397 treatment did not completely reverse the clinical manifestation of cGVHD, suggesting that additional and/or alternative cell types also contribute to these symptoms and the promotion of organ pathology in cGVHD mice. Indeed, others studies have implicated mast cells and eosinophils to also be involved in cGVHD-associated fibrosis [45, 46]. Moreover, although macrophage TGFβ is the key cytokine involved in fibroblast activation and collagen deposition in sclerodermatous cGVHD [27, 47], the production and deposition of autoantibodies in target tissues also leads to systemic fibrosis [48]. In our study, B cells were increased in the skin of cGVHD control mice but not changed by PLX3397 treatment. In the adult bone marrow, B-cell progenitors do not express the CSF-1R protein [49] and alongside our data, B-cell survival does not appear to be dependent upon CSF-1R signalling. Combinatorial experiments could also explore if combining PLX3397 treatment with B-cell-targeting drugs (such as B-cell-depleting antibodies, or the pharmacological B-cell inhibitor ibrutinib [50]) may more effectively alleviate and/or prevent cGVHD pathology. It would also be of interest in that regard to explore whether PLX3397 treatment similarly confers benefits in other, autoantibody-mediated mouse models of cGVHD.

### Pre-treatment with PLX3397 before the onset of cGVHD does not change disease progression or outcomes

As PLX3397 treatment during the onset of cGVHD did ameliorate but not completely annul disease symptoms, we also investigated whether depletion of macrophages/microglia immediately post-transplant could fully mitigate the development of GVHD altogether. The choice of our model system still results in successful chimerism [19] and using a low dose of donor T cells in the graft enabled us to study the effect of PLX3397 on mice that initially experience low grade aGVHD and then gradually progress into the cGVHD [20]. This is important, because our model mimics the clinical progression of the disease as a continuum from the acute to the chronic phase, allowing us to study the impact of PLX3397 as the disease evolves with time [2]. Strikingly, we found that PLX3397 treatment immediately after the transplant and during the initiation phase of aGVHD did not alter disease-specific outcomes. These findings suggest that macrophages and microglia are not necessarily critical for GVHD onset and/or during aGVHD initiation phase. Rather than (or in addition to) macrophage-driven inflammation and associated fibrosis as seen during cGVHD, GVHD onset (acute followed by chronic) may thus be driven by alloreactive T-cell-dependent cytokines [32], or by B cells and B-cell-secreted antibodies [48, 51] independent of macrophages. Of note, recipient (host) macrophages that survived the transplant conditioning regimen have also been implicated in shaping allogeneic donor T-cell response and, interestingly, their presence limits GVHD severity after allogeneic BM transplantation [52]. Future studies could examine to what extent early CSF1R inhibition with PLX3397 may have interfered with this.

### PLX3397 treatment in cGVHD attenuates cognitive impairment and neuroinflammation

We recently demonstrated infiltration of MHC II-expressing monocytes/macrophages and generalized microglial activation in cGVHD-affected mice [13]. We further showed that these infiltrating peripheral donor macrophages contribute to the behavioural dysfunction and neuroinflammatory changes observed during cGVHD. Others have also implicated microglia as key drivers of brain pathology in GVHD [53]. However, the specific involvement of both cell types in driving cGVHD pathology in the CNS had remained unclear. Here, we showed that PLX3397 treatment (days 30–70 post-transplant), which depletes both microglia and macrophages, attenuates spatial learning and memory deficits in cGVHD mice. Post-mortem studies further revealed that any residual IBA1^pos^ cells in PLX3397-treated mice also displayed significantly reduced MHC II expression, suggesting a reduced capacity for antigen presentation. Although we did not examine the lymphocytic infiltrate in the brains of cGVHD mice, these findings are of interest as PLX3397 treatment did attenuate cGVHD-associated increases in T-cell presence in peripheral target organs, such as the skin. Overall, these findings lend support to a pathogenic role for both donor macrophages and microglia in the cGVHD setting, although it remains to be elucidated whether these myeloid cell populations act independently or in conjunction with T cells to induce pathology. The latter is most likely, however, considering that coactivation of both cell types is required to promote disease progression in humanised cGVHD mice [54]. Finally, our study did not disentangle the individual cell-type-specific contributions of infiltrating donor macrophages and activated host microglia to cGVHD pathology. Although perhaps less relevant for translation, this topic thus remains open for investigation. Future studies that more specifically target peripheral myeloid subpopulations and spare microglia, for example, using CSF-1R blocking antibody M279 that is meant to be blood brain barrier impermeable^7^, may help resolve this issue.

In certain neuroinflammatory conditions, synapse loss is directly related to cognitive decline [55] and biomarkers that can non-invasively measure neurodegeneration are, therefore, useful to predict and/or monitor brain pathology and cognitive dysfunction early in the disease phase [56]. We previously reported a disruption to pre-synaptic compartment in cGVHD mice and speculated that this loss of synapses contributes to the cognitive dysfunction that is displayed by cGVHD mice [13]. Microglia are known to actively engulf and remove synapses in both the developing and adult brain [57–61]. Under neuroinflammatory conditions, activated microglia similarly and excessively prune synapses through the C1q complement pathway, which was functionally associated with cognitive decline [62–64]. Considering we have previously observed synaptic disruption in cGVHD mice [13], it is reasonable to speculate that the removal of microglia (and macrophages) with PLX3397 may have preserved synaptic loss and that this in turn precipitated in the improved cognitive performance of cGVHD mice receiving this treatment. However, more detailed follow-up studies and histological analyses would be required to explore whether there is reduced complement protein deposition in cGVHD brain tissue, and equivocally show that there is indeed a preservation of synapses in this pathology with PLX3397 treatment.

## Conclusions

Our study demonstrates the therapeutic potential of PLX3397 in improving clinical outcomes and reducing macrophage-driven peripheral organ pathology in cGVHD. We further show that these benefits of PLX3397 treatment extend to the CNS, with cognitive improvement and reduced neuroinflammation in cGVHD brains. Given the multifactorial nature of cGVHD, we posit that combinatorial approaches to treatment will improve disease outcomes with greater efficacy than current stand-alone methods. The present results identify PLX3397 as an effective drug to target specific aspects of cGVHD pathology, and our findings thus provide a basis for its inclusion in future clinical trials.

### Supplementary Information

**Additional file 1: Figure S1.** Histopathological assessment of cGVHD target organs and quantification of B cells and neutrophils in the skin with PLX3397 treatment during disease development (days 30–70 post-transplant). (A) Experimental timeline for irradiation, transplant and subsequent PLX3397 treatment. (B,C) Quantification of the IBA1^pos^ immunoreactive area (B) and CD68^pos^ immunoreactive area in liver tissue from syngeneic and cGVHD mice, with and without PLX3397 pre-treatment. Asterisks indicate post-hoc results. (D,E) Histopathology scores (D) and representative images of skin tissue stained with haemotoxylin and eosin (E) from syngeneic and cGVHD mice, with and without PLX3397 treatment. Arrowheads indicate separation between the dermal and epidermal layers as an example indication of pathology (see also Table 2). Asterisks indicate post-hoc results. (F,G) Histopathology scores (F) and representative H&E images (G) from liver tissue of syngeneic transplant controls and cGVHD mice, with and without PLX3397 treatment. (H,I) Histopathology scores (H) and representative H&E images (I) from lung tissue of syngeneic transplant controls and cGVHD mice, with and without PLX3397 treatment. (J,K) Histopathology scores (J) and representative H&E images (K) from gut tissue of syngeneic transplant controls and cGVHD mice, with and without PLX3397 treatment. (L) Quantification of B220^pos^ B cells in the epidermal layer of the skin tissue from syngeneic transplant controls and cGVHD mice, with and without PLX3397 treatment. (M) Quantification of Ly6B.2^pos^ neutrophils in the dermal layer of the skin tissue from syngeneic transplant controls and cGVHD mice, with and without PLX3397 treatment. Scale bar (E,G,I,K): 100 µm. Statistics: two-way ANOVA (B–D, F, H, J, L, M) followed by Bonferroni post hoc comparison. (E, G, I, K) Arrowheads point at GVHD pathology. Dots represent individual mice. *p < 0.05, ***p < 0.001, ****p < 0.0001.

**Additional file 2: Figure S2.** Histopathological assessment of cGVHD target organs liver, lung, and gut after pre-treatment with PLX3397 (days 0–30 post-transplant). (A) Experimental timeline for irradiation, transplant and subsequent PLX3397 treatment. (B, C) Histopathology scores (B) and representative H&E images (C) from liver tissue of syngeneic transplant controls and cGVHD mice, with and without PLX3397 treatment. (D, E) Histopathology scores (D) and representative H&E images (E) images from lung tissue of syngeneic transplant controls and cGVHD mice, with and without PLX3397 treatment. (F, G) Histopathology scores (F) and representative H&E images (G) from gut tissue of syngeneic and cGVHD mice, with and without PLX3397 treatment. Scale bar (C, E, G): 100 µm. Statistics: one-way ANOVA (A–G) followed by Bonferroni post hoc comparison. Arrowheads indicate evidence of pathology (B,D,F). Dots represent individual mice. ****P < 0.0001.

## Data Availability

All data generated or analysed during this study are included in this published article [and its supplementary information files].
